# Characteristics and management of bone and joint tuberculosis in native and migrant population in Shanghai during 2011 to 2015

**DOI:** 10.1186/s12879-018-3456-3

**Published:** 2018-11-01

**Authors:** Yun Qian, Qixin Han, Wenjun Liu, Wei-En Yuan, Cunyi Fan

**Affiliations:** 1 Department of Orthopedics, Shanghai Jiao Tong University Affiliated Sixth People’s Hospital, 600 Yishan Road, Shanghai, 200233 People’s Republic of China; 2 Center for Reproductive Medicine, Renji Hospital, School of Medicine, Shanghai Jiao Tong University, Shanghai, 200135 China; 3 Shanghai Key Laboratory for Assisted Reproduction and Reproductive Genetics, Shanghai, 200135 China; 4 Department of Orthopedics, Shanghai Sixth People’s Hospital East Affiliated to Shanghai University of Medicine & Health Sciences, Shanghai, 201306 China; 5 Taishan Medical University, Taian, 271016 China; 6 Engineering Research Center of Cell & Therapeutic Antibody, Ministry of Education, and School of Pharmacy, Shanghai Jiao Tong University, Shanghai, 200240 China

**Keywords:** Bone and joint tuberculosis, Native, Migrant, Surgery, Anti-tuberculosis, Shanghai

## Abstract

**Background:**

China had the third highest burden of tuberculosis population in the world. Bone and joint tuberculosis was a major part and its characteristics were rarely discussed before. This study was designed to review the characteristics and management of bone and joint tuberculosis among native and migrant population in Shanghai, China during 2011–2015.

**Methods:**

A retrospective analysis of the patient clinical records on their demographic information, clinical features and treatment was conducted from three tertiary referral hospitals. Analysis of continuous variables included calculation of the median value with interquartile range. Categorical variables were displayed as percentages and compared using the Fisher’s exact test and chi-square test. All continuous variables were compared using Student’s unpaired t-test and Mann Whitney U test.

**Results:**

One hundred fifteen patients with bone and joint tuberculosis were involved in this study. Native people were generally older (*p* = 0.003) and had more comorbidities like hypertension (40.74% vs. 16.39%, *p* = 0.004), diabetes mellitus (38.89% vs. 13.11%, *p* = 0.001), and cancer (31.48% vs. 14.75%, *p* = 0.032) than migrants. Migrant patients generally experienced a longer period of uncomfortable feelings before going to doctor than native people (*p* = 0.007). Spine was a major infection site in comparison with other peripheral joints. Radiological evaluation displayed increased osteolytic reaction in migrant patients compared with native people (*p* = 0.031). The mean time for anti-tuberculosis treatment was significantly longer in native Shanghai patients (8.96 months vs. 7.94 months, *p* = 0.003). The curative ratio displayed a significant difference between native and migrant patients (88.24%vs.75.93%, *p* = 0.009).

**Conclusion:**

Bone and joint tuberculosis exhibited a poorer outcome in migrant people, who also had longer period of manifestation, more severe osteolytic reaction from CT scan and higher recurrent rate than native people. The surgical treatment in addition to anti-tuberculosis drug therapy had great implications for bone and joint tuberculosis recovery.

## Background

In 2015, new tuberculosis cases were estimated to reach 10.4 million in the whole world, and six nations had around 60% of all incidences, among which were India, Indonesia, China, Nigeria, Pakistan and South Africa [1]. China had the third highest burden of tuberculosis population in the world, with approximately 0.93 million new cases in 2015, according to the statistics released by National Health and Family Planning Commission of China [2].

The bone and joint tuberculosis (BJTB) represents approximately 2–5% in all TB cases, and 10% in extra-pulmonary tuberculosis (EPTB), ranking the third common location of all TB infection [3]. Osteoarticular involvement primarily contains spine, shoulder, elbow, wrist, hip, knee, ankle and foot [4, 5]. Although great efforts have been made to control this serious public health issue, very limited achievements were made even in major metropolises in China due to many migrant workers from less developed regions and failure to successful diagnosis with atypical presentation. Besides, classic drug therapy is not really efficacious for those patients who are at advanced stages with specific joint activity restriction. The efficient treatment calls for a combination of surgical removal and drug therapy [6].

There is very limited published literature on the epidemiology, clinical features and diagnosis, as well as treatment of BJTB in Shanghai, the most prosperous city in China with the most complicated population proportion. In this study, BJTB was comprehensively evaluated based on data from three tertiary referral centers in Shanghai from 2011 to 2015, including their demographic information, clinical features and treatment.

## Methods

All data for BJTB cases were collected from the medical history system and database from three tertiary referral hospitals in Shanghai from the year 2011 to 2015. In this study, patients were classified into native Shanghai people group and migrant group. Migrants are defined as floating population who leave their birthplaces and live in another city without local identity. Native people are considered as resident population who live and have local residency [7]. We defined TB patients using laboratory examinations, radiographs and biopsies. The patient records included their demographic features, duration of disease onset, clinical manifestation and symptoms, concomitant diseases, laboratory and radiological examination, treatment and follow-up. Part of the patients’ medical documents which were incomplete or incorrect were removed from this study. The suggestive laboratory tests involve neutrophils, erythrocyte sedimentation rate (ESR), C-reactive protein (CRP), hemoglobin, T-SPOT, and sputum culture. Relevant radiological examination includes chest X-ray, diseased bone or joint X-ray, computed tomography (CT) scan or magnetic resonance imaging (MRI). For different stages of bone and joint tuberculosis, we treated patients with different procedures, either conservative medication therapy and minimally invasive practices or open surgeries. The relative standards for open surgical treatment include advanced stage of TB, major limitations in bone and joint activity, and patients’ willingness to accept surgeries. Tubercular masses and regional lesions were removed for pathological evaluation. Besides, the open surgery provided us with clear vision and possibly full recovery of joint motion. However, extended clearance of surrounding tissues and bones were not recommended in order not to spread the pathogen. In those severe cases with vast devastation of structures, osteotomy and orthopedic operations were also required. For spine TB patients, spinal fusion was adopted for reconstruction of stable joints via a direct lateral interbody fusion manner using minimal invasive operations. For those who experienced stiff joint and limitations in activity, joint arthrolysis was carried out basically including capsule release, muscle and tendon repair as well as bone reconstruction [8].Biopsy of bone or soft tissue was conducted in partial patients.

For peripheral BJTB patients who accepted surgeries, they needed to follow passive and active joint motion practice. For instance, elbow TB patients were required to regain elbow range of motion by extension of 0° and flexion of 120°. At the same time, celecoxib or indomethacin were given to patients for oral intake thrice a day for heterotopic ossification prevention. Proper antibiotic therapy was used in all patients.

### Statistical analysis

Analysis of continuous variables included calculation of the median value with interquartile range. Categorical variables were displayed as percentages and compared using the Fisher’s exact test and chi-square test. All continuous variables were compared using Student’s unpaired t-test and Mann Whitney U test. A *p* value less than 0.05 was considered statistical significance.

## Results

### Epidemiological analysis

**Fig. 1:**
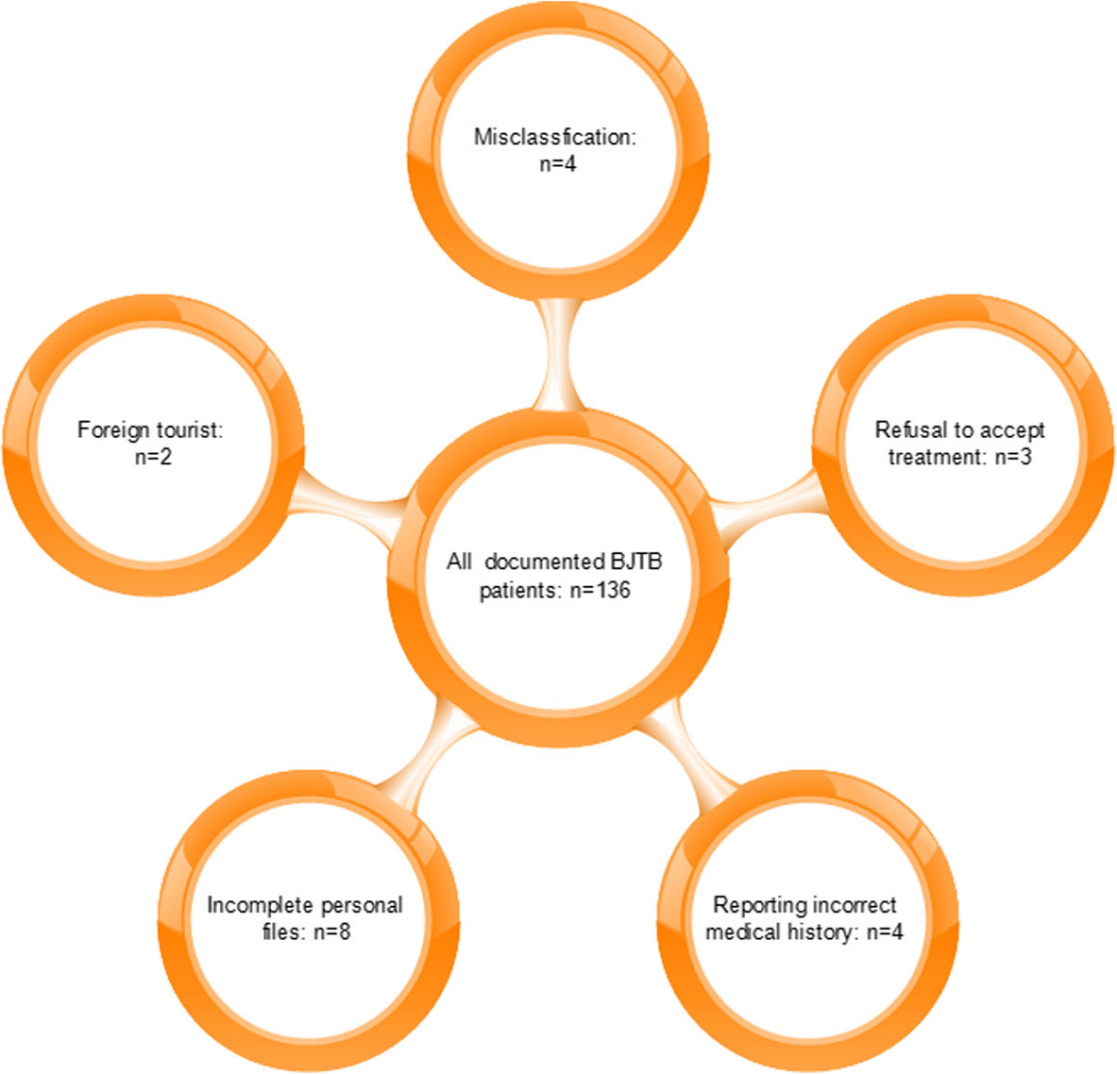
Schematic structure of patient enrollment

**Fig. 2:**
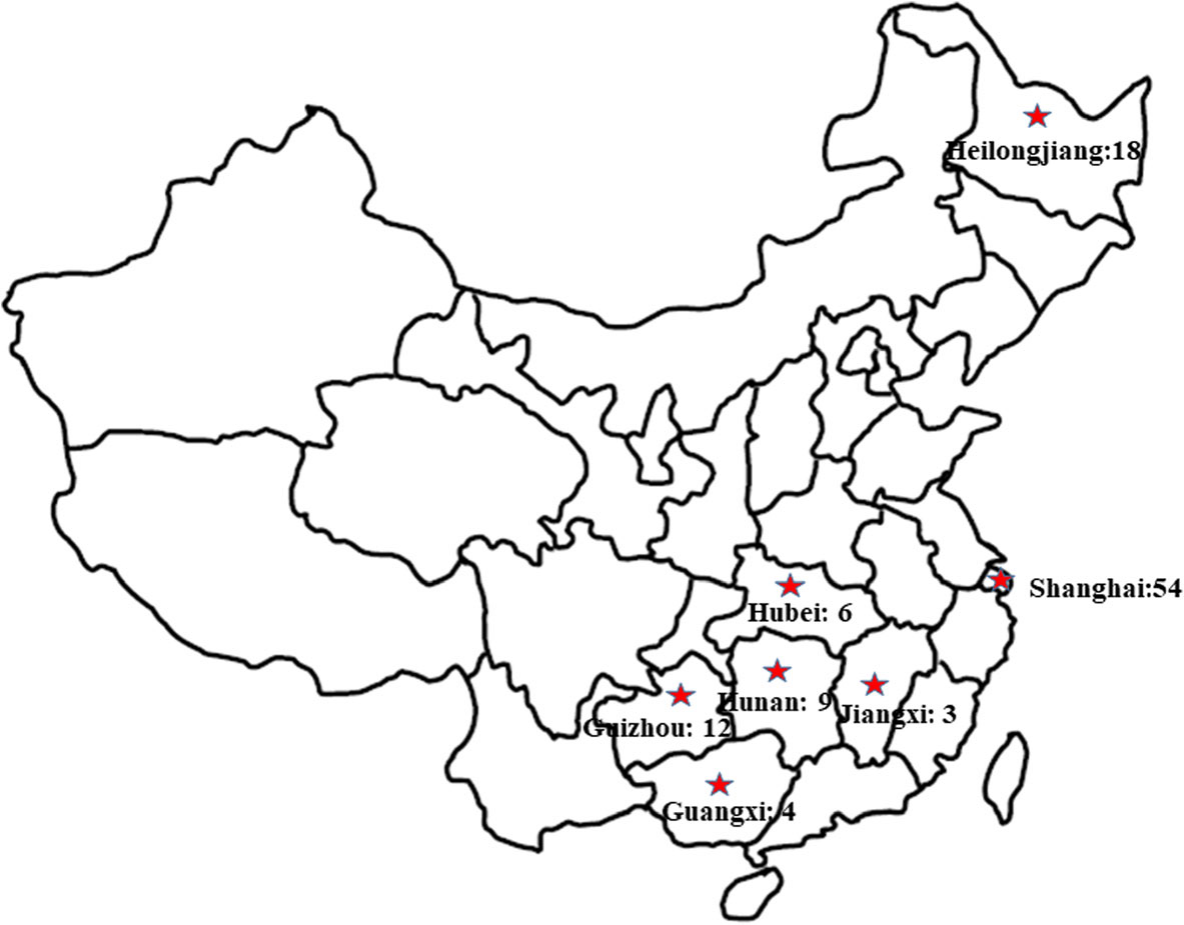
The map showing the study area and sample size

After a general examination of all patients’ medical documents, a total of 136 patients were diagnosed with bone and joint tuberculosis, among which 115 patients were included in this study (Fig. 1). Of all the patients, 54 patients were native Shanghai people, while 61 were migrant from other provinces in China. Among these migrants, 18 were from Heilongjiang, and the second largest immigration came from Guizhou with 12 patients, followed by Hunan (9), Hubei (6), Guangxi (4), and Jiangxi (3) (Fig. 2). The male/female ratio was 37/17 and 42/19 in native people and migrants respectively. The median age of these patients was 55.89, ranging from 25 to 83 in native people. However, the mean age was 46.84 in migrants, varying from 16 to 85 (*p* = 0.003). The curative ratio displayed a significant difference between native people and migrants, with 45 out of 51 (88.24%) and 41 out of 54 (75.93%) (*p* = 0.009). In those recurrent cases, there was 1 death case (1.96%) in native people within 1 year from initial diagnosis. In contrast, 4 migrant patients (7.41%) died during the same period of time. The general demographic data were displayed in Table 1 according to population classification.

**Table 1:** Demographic information of bone and joint tuberculosis patients from 2011 to 2015 in Shanghai

Demographic information	Native people	Migrant people	*P* value
Patients	54	61	/
Male/female	37/17	42/19	0.969
Age	55.89(25–83)	46.84(16–85)	0.003^a^
Recurrent(Alive)	5(9.80%)	9(16.67%)	0.009^b^
Dead	1(1.96%)	4(7.41%)	
Cured	45(88.24%)	41(75.93%)	

^a^Man whitney U test

^b^Fischer Exact test

### Clinical evaluation

**Fig. 3:**
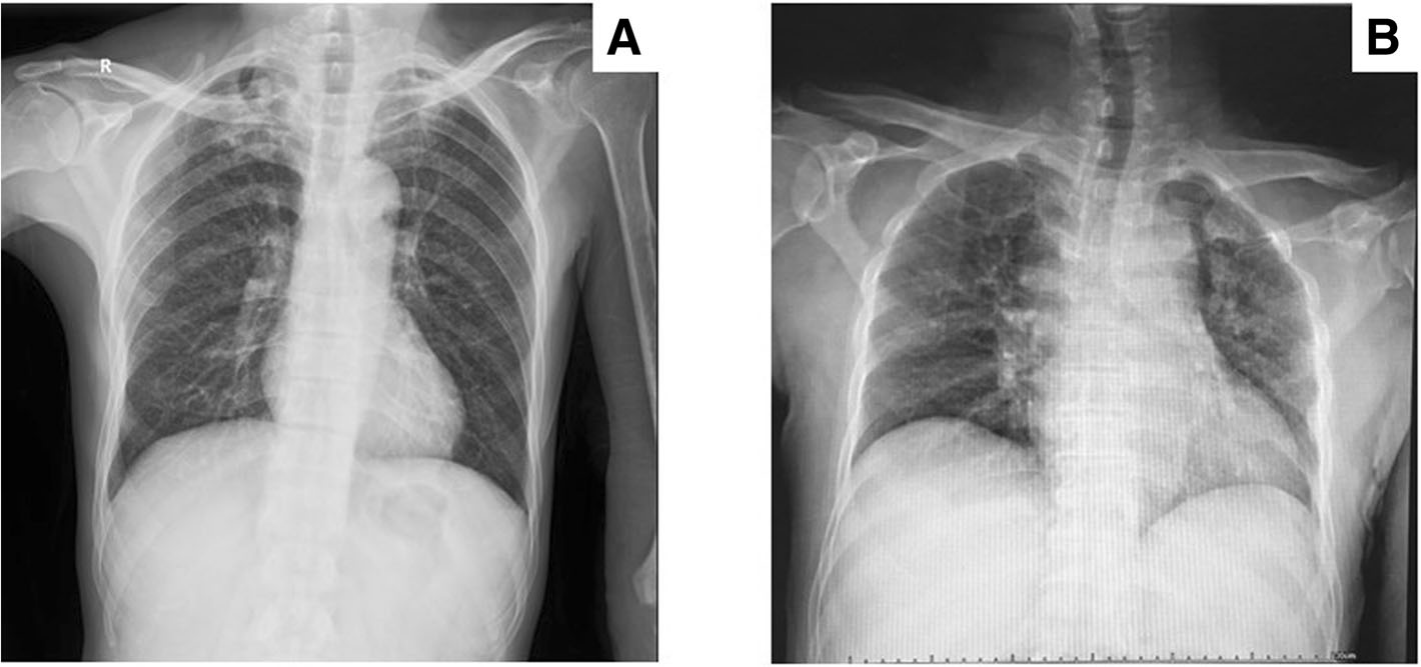
X-ray of pulmonary tuberculosis. **a** Moderate infection; **b** Severe infection

**Fig. 4:**
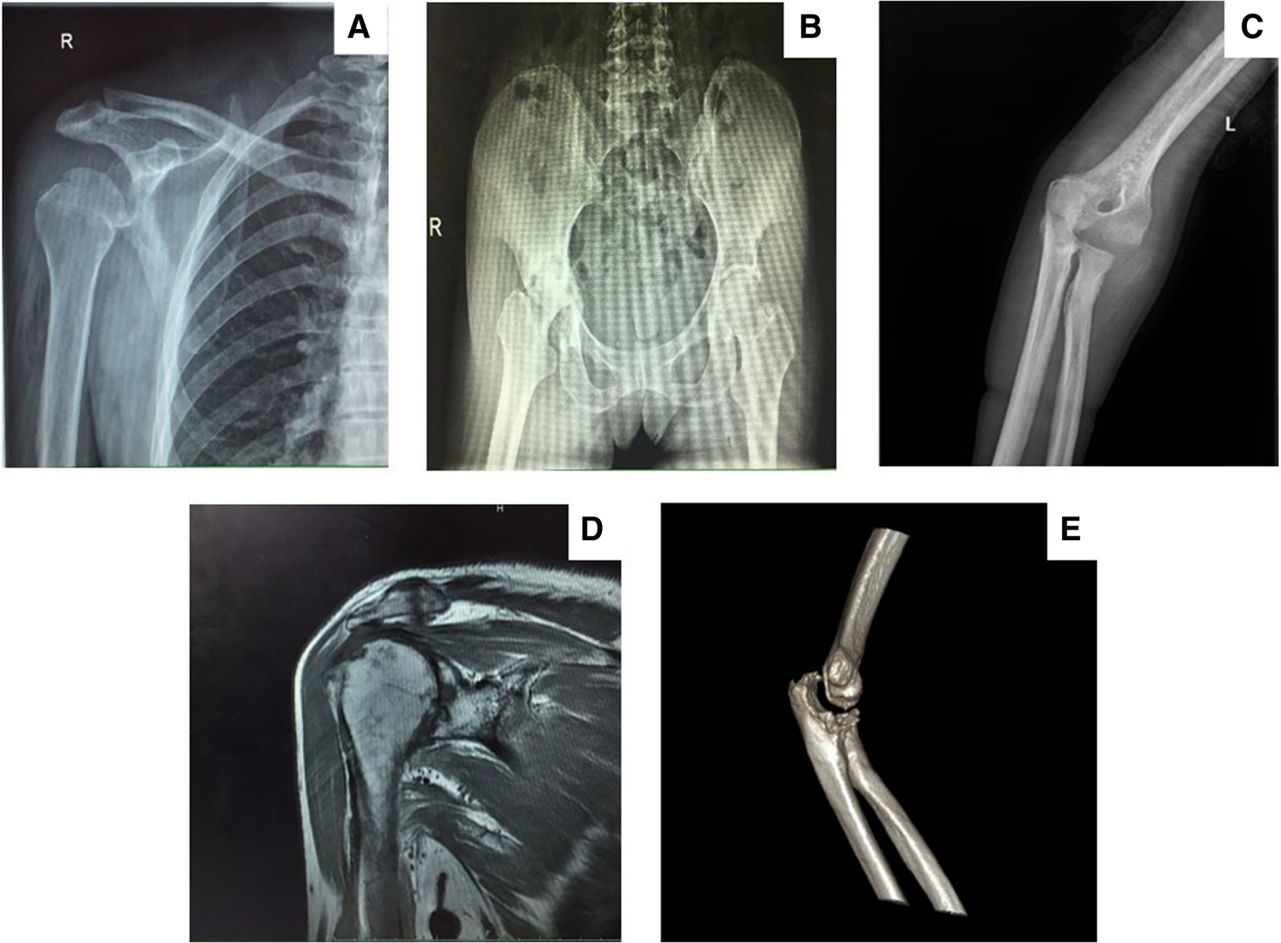
X-ray, CT and MR of different peripheral bone and joint tuberculosis. **a** X-ray of shoulder TB; **b** X-ray of hip TB; **c** X-ray of elbow TB; **d** MR of shoulder TB; **e** CT of elbow TB

**Fig. 5:**
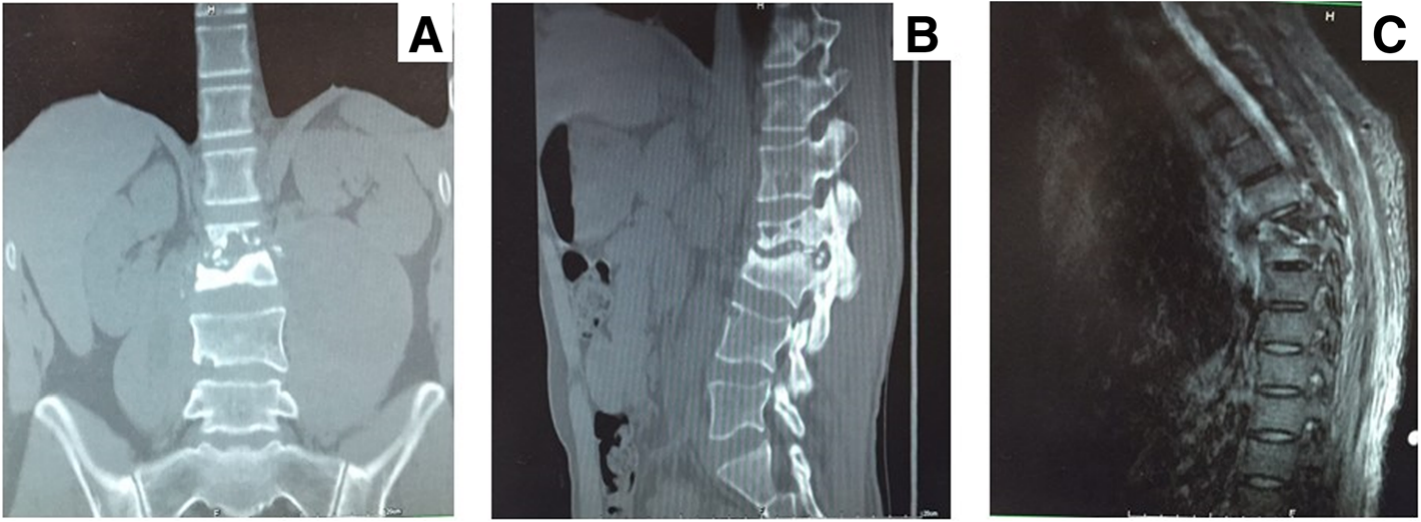
CT and MR of spine tuberculosis. **a** CT of spine TB: anterioposterior position; **b** CT of spine TB: lateral position; **c** MR of spine TB

Clinical manifestation and presentation of BJTB patients normally involved pain (92.59% vs. 95.08%), night sweat (27.78% vs. 19.67%), fever (44.44% vs. 40.98%), cough (12.96% vs. 18.03%), weight loss (22.22% vs. 16.39%), paraplegia (5.56% vs. 14.75%), and limitations in activity (42.59% vs. 31.15%) in native and migrant patients respectively, with a major complaint in pain at infection site. It did not show any significant difference between people from Shanghai and other provinces (Table 2). The average duration of symptom onset to hospital was 5.63 months, varying from 1 to 72 months in native Shanghai patients. Nevertheless, migrants experienced uncomfortable feelings and symptoms for a much longer period, with an average duration of 8.57 months, varying from 1 to 36 months (*p* = 0.007). Partial peripheral joint infection patients experienced limitations in joint range of motion (ROM), especially in elbow, hip, shoulder and knee TB infection. Some patients had comorbidities, including hypertension (HT, 40.74% vs. 16.39%), diabetes mellitus (DM, 38.89% vs. 13.11%), Human Immunodeficiency Virus (HIV, 5.56% vs. 4.92%), Rheumatoid Arthritis (RA, 11.11% vs. 8.20%), syphilis (5.56% vs. 3.28%), previous tuberculosis (9.26% vs. 11.48%) and cancer (31.48% vs. 14.75%) in two groups (Table 2). Migrants were younger and had less concomitant diseases than native patients. BJTB accompanied by pulmonary tuberculosis was noticed (40.74% vs. 44.26%), while other types of EPTB were also found, including lymph nodes (3.70% vs. 4.92%) and urinary tract and kidney (3.70% vs. 8.20%). There was no notable difference between native people and migrants either (*p* = 0.888). For BJTB types, there were 75 spinal tuberculosis cases, and the other types contained knee (11), hip (10), shoulder (7), elbow (6), ankle/foot (4) and wrist (2). In spine tuberculosis, lumbar infection was the most common (32). Thoracic and cervical vertebra infection was next to it, with 24 and 16 cases respectively, followed by paravertebral abscess (3). No cases were reported to have over one infection location. Migrant-dependent details were displayed in Table 2. No significant differences were found in laboratory examination or drug resistance patterns between groups (Table 2). Twenty-one patients were reported severe infection findings, like osteolytic reaction in native Shanghai people, compared with 36 in migrants in CT scan (*p* = 0.031), in spite of insignificant differences in other radiological examinations (Figs. 3, 4, 5).

**Table 2:** Clinical evaluation of bone and joint tuberculosis patients from 2011 to 2015 in Shanghai

Clinical features			Native people	Migrant people	*P* value
Duration of symptom onset to hospital			5.63(1–72)	8.57(1–36)	0.007^a^
Symptom	Pain		50(92.59%)	58(95.08%)	0.705^b^
	Night sweat		15(27.78%)	12(19.67%)	0.306
	Fever		24(44.44%)	25(40.98%)	0.708
	Cough		7(12.96%)	11(18.03%)	0.455
	Weight loss		12(22.22%)	10(16.39%)	0.428
	Paraplegia		3(5.56%)	9(14.75%)	0.107
	Limitations in activity		23(42.59%)	19(31.15%)	0.203
Laboratory exam	Neutrophils (%)		61.57(39.10–79.40)	62.40(42.40–79.60)	0.659
	ESR(mm/h)		17.51(7.60–25.90)	16.75(7.50–25.20)	0.346
	CRP(mg/l)		13.57(9.22–20.22)	13.13(8.45–21.23)	0.358^a^
	Hemoglobin(g/l)		111.98(86–143)	112.89(80–139)	0.733
	T-SPOT		24(64.86%)	25(44.64%)	0.056
	Sputum culture		31(73.81%)	35(71.43%)	0.800
	Biopsies		0	2	/
Radiological exam	Chest	negative	24(44.44%)	32(52.46%)	0.442
		moderate	23(42.59%)	19(31.15%)	
		severe	7(12.96%)	10(16.39%)	
	Joint	negative	0	0	0.031
		moderate	33(61.11%)	25(40.98%)	
		severe	21(38.89%)	36(59.02%)	
Infection site	Peripheral	Shoulder	3(5.56%)	4(6.56%)	0.639
		Elbow	3(5.56%)	3(4.92%)	
		Wrist	1(1.85%)	1(1.64%)	
		Hip	4(7.41%)	6(9.84%)	
		Knee	8(14.81%)	3(4.92%)	
		Ankle/foot	3(5.56%)	1(1.64%)	
	Spine	Cervical	7(12.96%)	9(14.75%)	0.649
		Thoracic	8(14.81%)	16(26.23%)	
		Lumbar	16(29.63%)	16(26.23%)	
		Paravertebral abscess	1(1.85%)	2(3.28%)	
Concomitant TB	Pulmonary		22(40.74%)	27(44.26%)	0.888
	Lymph nodes		2(3.70%)	3(4.92%)	
	Urinary tract and kidney		2(3.70%)	5(8.20%)	
Comorbidity	HT		22(40.74%)	10(16.39%)	0.004
	DM		21(38.89%)	8(13.11%)	0.001
	RA		6(11.11%)	5(8.20%)	0.596
	HIV		3(5.56%)	3(4.92%)	1.000
	Syphilis		3(5.56%)	2(3.28%)	0.664
	Previous TB		5(9.26%)	7(11.48%)	0.698
	Cancer		17(31.48%)	9(14.75%)	0.032
Drug resistant pattern	Isoniazid		14(25.93%)	18(29.51%)	0.669^c^
	Rifampin		8(14.81%)	11(18.03%)	0.643^c^
	Ethambutol		6(11.11%)	8(13.11%)	0.743^c^
	Streptomycin		10(18.52%)	11(18.03%)	0.946^c^
	Multidrug resistance		0	2(3.28%)	0.497^b^
Surgical intervention			34(62.96%)	32(52.46%)	0.256
Infection			5(9.26%)	8(13.11%)	0.515
Drug			40(74.08%)	46(75.41%)	0.869
Mean time for anti-tuberculosis treatment			8.96(6–12) ^d1^	7.94(4–12) ^d2^	0.003
Follow-up duration			15.16(12–24)^d1^	13.63(12–23)^d2^	0.011^a^

*ESR* erythrocyte sedimentation rate, *CRP* C-reactive protein, *HT* hypertension, *DM* diabetes mellitus, *RA* rheumatoid arthritis, *HIV* Human Immunodeficiency Virus, *TB* tuberculosis

^a^Mann Whitney U test

^b^Fischer Exact test

^c^Pearson chi square test

^d1^3 patients were lost in the follow-up

^d2^7 patients were lost in the follow-up

The median time for anti-tuberculosis treatment showed a significant difference between two groups. It was 8.96 months in native Shanghai patients compared with 7.94 months in migrant patients (*p* = 0.003). The treatment outcome at 1 year (including dead cases within 1 year) was achieved in 51 of 54 native patients and 54 of 61 migrants. The average follow-up time was 15.16 months and 13.63 months in native and migrant groups respectively (*p* = 0.011). Ten patients were lost in follow-up or unwilling to report their conditions. Curative ratio was 88.24% (45 out of 51) in native patients and 75.93% (41 out of 54) in immigration patients.

## Discussion

Tuberculosis is generally considered as an economic and sanitary condition-related infectious disease, with high prevalence in developing countries [9]. Bone and joint tuberculosis has a relatively lower incidence. Shanghai is a major metropolis in the world, which has a comparable economic and health care level with Chicago, Seoul, Hong Kong, Singapore, and so forth [10]. Besides, established health care system has enabled people to enjoy a higher quality of life in Shanghai [11]. Even under this environment, the overall BJTB incidence is still much higher than it in other big cities, like Paris [12]. It is estimated that the complex population proportion and migration from underdeveloped provinces are major causes for tuberculosis incidences [13]. The statistics showed that older people were more prone to being infected with BJTB than young people from native group. And the majority of infected migrants were younger than native people, which probably resulted from their status of migrant workers. The age-related characteristics were also observed in other researches [14, 15]. This study was based on the BJTB cases occurring between the year 2011 and 2015, during which time sick immigration patients were mainly from several provinces of high TB incidences, like Heilongjiang, Guizhou, Hunan, Hubei, Guangxi, and Jiangxi. These areas have occupied the top places in the list of TB incidence in China for many years due to low economic and health care development [16]. Although the overall infected patients were declining from 2011 to 2015 by year, the proportion of migrants was increased every year [17]. Besides, as for the types of BJTB cases, spinal TB was a leading type [18]. Migrants had a higher incidence of spinal TB, accounting for 70.49%. It is consistent to the data of other places reporting BJTB cases, like United Kingdom 92% [19], Holland 66% [20], and France 68% [21]. To evaluate the difference between two population groups, we decided to follow a population classification based analysis in the following data. The mean duration between disease onset and clinical treatment was 5.63 and 8.57 months in two groups respectively. This was much shorter than a report conducted in Iran, with 12.94 months in average (1 to 108 months) [22], and relatively longer than that from Denmark research [23].

The comorbidities like DM, HT, and cancer could compromise the immune system of these patients, leading to their vulnerable exposure to TB pathogen and BJTB onset. The clinical manifestation generally was characteristic of TB pathogen infection, and pain was the most common symptom among all, which originated from bone erosion and nerve irritation. This result was much higher than that from Western countries with neurological complaints varying from 20 to 50% [23]. Other symptoms like cough, fever, and sweat were related to respiratory system infection. BJTB accompanied pulmonary TB was 40.74% and 44.26% in native and migrant groups respectively in this study, which was higher than the average data from developed regions in the world, ranging from 23 to 35% [11].

As is known to all, only a small portion of the laboratory examination has direct relationship with TB diagnosis, like T-SPOT and sputum culture as well as pathological examination. All the patients had at least one positive result from the above three tests to confirm TB infection, in addition to radiological findings as well as clinical presentation. As tuberculosis is getting increasingly resistant to drug, the anti-tuberculosis drug resistant pattern was evaluated as well for proper drug selection. Many patients displayed monoresistance to a single drug. Two patients from migrant group experienced multidrug resistant. One patient had previous TB history. The other patient had concomitant kidney tuberculosis. It was believed that their multidrug resistant condition was also because they received irregular anti-tuberculosis treatment before tuberculosis diagnosis. This is in consistent with the conclusion of a previous research. Chen and colleagues conducted a nationwide BJTB research and collected 113 cases for bacterial evaluation. They discovered 17 multidrug resistance cases and among them, 2 cases were extremely severe resistant. Like our patients, those people had either concomitant tuberculosis, past tuberculosis infection or irregular anti-tuberculosis treatment before final diagnosis [24, 25]. The radiological examination is of vital importance for supplementary diagnosis. In X-rays, bone defects were noticed. CT provided us with a three dimensional structure of bone erosion. Moreover, MRI offered a more comprehensive presentation of osteoarthritis changes, with bone devastation, mass edema and abscess formation. It was mainly applied in spinal TB patients. Correct and timely diagnosis is of great significance to final treatment and recovery for BJTB patients.

Surgical treatment remained a primary procedure for BJTB in this study. Although BJTB patients sometimes had pulmonary TB at the same time, early symptoms were generally atypical and they went for medical treatment at an advanced level. Because the structure of bone and joints was severely damaged by TB pathogen, simple anti-tuberculosis drugs were unable to restore functional activity. Moreover, the erosive remnants were at high risk of further collapsing and deformation. Therefore, proper surgical intervention was extremely necessary, including arthroplasty and arthrolysis. Joint arthrolysis typically fit those cases with limited joint range of motion [26, 27]. Arthroplasty was more appropriate for complete devastation of joint structure, with limited choices of reconstruction except artificial joint replacement [28]. For spine TB patients, they also received intervertebral disc fusion via minimal invasive operations [29]. In this study, 66 patients received joint arthrolysis and no arthroplasty was performed. These patients received anti-tuberculosis drugs just like those who received conservative therapy. In addition, postoperative active and passive exercise was of vital significance to functional recovery for patients, for instance, in elbow stiffness [30]. Celecoxib or indomethocin was taken for prevention of heterotopic ossification occurrence.

Standard anti-tuberculosis therapy was initiated in partial patients immediately after surgery. In contrast, other patients were recommended to start their anti-tuberculosis treatment at specialized infectious disease department or hospital. They needed systematic evaluation of anti-tuberculosis treatment from professionals of infectious diseases. In China, we suggest the entire treatment should last for 6 to 12 months, which is similar to common practices in Western world [31]. However, the mean time for anti-tuberculosis treatment showed a significant difference between two groups. Migrant patients generally received anti-tuberculosis drugs irregularly for a shorter period of time.

The general treatment success rate was 81.90% in all patients, with 88.24% in native patients and 75.93% in migrants. The relatively higher recurrent rate and poorer outcome in migrants were estimated due to their migrant characteristics, disobedience to self-supervision on anti-tuberculosis drugs intake as well as failure in receiving medical treatment because of heavy economic burden [17]. These people migrated frequently due to job insecurity. Therefore, they tended to overlook taking medication regularly. Meanwhile, in our long-term follow-ups, some of migrant patients considered medication was insignificant since they regained functional joint activity after surgery. These people refused to supervise themselves on anti-tuberculosis drugs intake. More importantly, the poor outcomes also resulted from huge economic burden on migrant patients because many of them were not covered by local medical insurance. Insufficient government budget outlay in public health, especially for severe infectious diseases, is a common problem in developing countries. In China, TB patients need to cover 30–44% of total medical expense by themselves. It means more for migrants. However, the general successful result was already close to that from developed countries in Europe, like Denmark [23]. Johansen and colleagues retrospectively reviewed 282 cases of bone and joint tuberculosis in 17 years. Their general success rate was 83%. However, 15 patients died within 1 year of follow-up in their study. Besides, only 44% of all BJTB patients received open surgeries because they did not consider surgery as a major contributing factor for final successful treatment. It was especially valuable in such a big city with over 24 million people and highly complicated population proportion. The population mobility was used to evaluate the attraction of a major city, which also may bring about potential risks in transmitting infectious diseases [32]. This was one important reason proposed previously for the high incidence of BJTB in Shanghai. However, experienced medical team and sophisticated treatment protocol as well as timely diagnosis and treatment has contributed to successful rehabilitation in BJTB patients. More emphasis on medical care reforms will improve the overall tuberculosis treatment in Shanghai and nationwide.

## Conclusion

BJTB was not uncommon in large developed cities, like Shanghai. The migrants had a higher risk of recurrence and exhibited a relatively poorer outcome than native patients. The typical BJTB patients displayed bone defects and erosion at lesion site from X-ray, CT or MRI examination. The diagnosis could be assisted by T-SPOT, sputum culture and biopsy. The surgical intervention and postoperative management was vital to restore functional joint activity and ensured final recovery.

## Abbreviations

BJTB: bone and joint tuberculosis
CRP: C-reactive protein
CT: computed tomography
DM: diabetes mellitus
EPTB: extra-pulmonary tuberculosis
ESR: erythrocyte sedimentation rate
HIV: Human Immunodeficiency Virus
HT: hypertension
MRI: magnetic resonance imaging
RA: Rheumatoid Arthritis
ROM: range of motion
UCLA: University of California, Los Angeles
